# Long-term mortality due to infection associated with elevated liver enzymes: a population-based cohort study

**DOI:** 10.1038/s41598-021-92033-1

**Published:** 2021-06-14

**Authors:** Tak Kyu Oh, Eun Sun Jang, In-Ae Song

**Affiliations:** 1 Department of Anesthesiology and Pain Medicine, Seoul National University Bundang Hospital, 166 Gumi-ro, Bundang-gu, Seongnam, 463-707 South Korea; 2 Department of Internal Medicine, Seoul National University Bundang Hospital, Seongnam, South Korea; 3 Department of Internal Medicine, College of Medicine, Seoul National University, Seoul, South Korea

**Keywords:** Diseases, Gastroenterology, Risk factors

## Abstract

We aimed to investigate whether elevated liver enzymes in the adult population were associated with mortality due to infection. As a population-based cohort study, data from the National Health Insurance Service Health Screening Cohort were used. Adult individuals (aged ≥ 40 years) who underwent standardized medical examination between 2002 and 2003 were included, and infectious mortality was defined as mortality due to infection between 2004 and 2015. Aspartate transaminase (AST), alanine aminotransferase (ALT), γ-glutamyl transpeptidase (γ-GTP), AST/ALT ratio, and dynamic AST/ALT ratio (dAAR) were included in multivariable Cox modeling. A total of 512,746 individuals were included in this study. Infectious mortality occurred in 2444 individuals (0.5%). In the multivariable model, moderate and severe elevation in AST was associated with 1.94-fold [hazard ratio (HR):1.94, 95% confidence interval (CI) 1.71–2.19; *P* < 0.001] and 3.93-fold (HR: 3.93, 95% CI 3.05–5.07; *P* < 0.001) higher infectious mortality respectively, compared with the normal AST group. Similar results were observed for moderate and severe elevation in ALT and mild, moderate, and severe elevation in γ-GTP. Additionally, a 1-point increase in the AST/ALT ratio and dAAR was associated with higher infection mortality. Elevated liver enzymes (AST, ALT, AST/ALT ratio, γ-GTP, and dAAR) were associated with increased infectious mortality.

## Introduction

Infectious disease is one of the most important causes of mortality and morbidity in human history^1^. Mortality and morbidity due to infectious diseases are common despite advances in medicine. Every year, the influenza virus infects 10–40% of the world population^2^. During the 2009–2010 H1N1 influenza pandemic in the United States, 59 million people were infected, 265,000 were hospitalized, and there were 12,000 deaths^3^. Currently, the world population is facing the coronavirus disease-2019 pandemic as a global public health crisis^4^.

The liver plays a critical role in host defense mechanisms during infection^5^. It contains many innate immune cells, such as macrophages, natural killer cells, natural killer T cells, and gamma-delta T cells, which contribute to the immunologic elimination of microorganisms by the endothelial cells and Kupffer cells of the liver^6^. The antigen-rich blood from the gastrointestinal tract passes through the liver, and the lymphocytes and natural killer T cells play critical roles as the first immune defense mechanism against invading pathogens^7^. Thus, impaired immunity due to liver dysfunction might lead to chronic infection and cancer^8^. Among common liver diseases, liver cirrhosis is considered an immunocompromised condition that increases the susceptibility to bacterial infection, resulting in a higher rate of mortality^9^. Similarly, abnormal liver function due to any liver disease can cause innate immune dysfunction^10^, resulting in a higher risk of infection and mortality due to infection. Liver enzymes have been widely used to evaluate and screen for abnormal liver function in the adult population^11–13^, and elevated liver enzymes might be related to a higher risk of mortality. However, the relationship between the elevation of liver enzymes and the risk of infection or infectious mortality has not been identified.

Therefore, this study aimed to investigate whether elevated liver enzymes were associated with a higher risk of infectious mortality among the adult population. Specifically, liver enzymes, such as aspartate transaminase (AST), alanine aminotransferase (ALT), and γ-glutamyl transpeptidase (γ-GTP) are evaluated during a standardized medical examination in South Korea.

## Methods

### Study design and ethical statements

This study involved human participants, and all procedures were conducted in accordance with the guidance provided by the relevant ethics boards. The Institutional Review Board (IRB) of Seoul National University Bundang Hospital (SNUBH) (X-1911-579-902) and the Health Insurance Review and Assessment Service (NHIS-2020-2-067) approved the study protocol. The requirement for informed consent was waived by the IRB of SNUBH because the data analyses were performed retrospectively, using anonymous data derived from the South Korean NHIS database.

### Data source: NHIS-HEALS database and the study population

The NHIS-National Health Screening Cohort (NHIS-HEALS) was used for this study^14^. As the sole public insurance system in South Korea, the NHIS collects information regarding demographics, socioeconomic status, diagnosis of diseases using the International Classification of Diseases, tenth revision (ICD)-10 codes, and treatment of the diseases. All disease diagnoses and prescription information of drugs and/or procedures should be registered in the NHIS database to receive financial support from the government, suggesting that the reliability of the NHIS database is sufficient. Subscribers to the NHIS who are ≥ 40 years of age are recommended to undergo a standardized medical examination every 2 years^15^. Using the results of the standardized medical examination, the NHIS constructed the NHIS-HEALS database for medical research. The cohort in this study comprised 514,795 individuals who underwent standardized medical examinations between 2002 and 2003 and were followed up until 2015. The database contains information regarding the body mass index (BMI), laboratory test results, including liver enzymes, and information acquired by administering questionnaires about lifestyle (exercise, alcohol consumption, and smoking). We included individuals who underwent a standardized medical examination between 2002 and 2003. Individuals who died between 2002 and 2003 or had missing liver enzymes data during this period were excluded from the analysis.

### Liver enzymes as main independent variables

AST, ALT, AST/ALT ratio, γ-GTP, and dynamic AST/ALT ratio (dAAR) were measured from the venous blood sample in the standardized medical examinations between 2002 and 2003. Based on the recent guidelines regarding the evaluation of abnormal liver chemistry^16^, the AST, ALT, and γ-GTP levels were categorized into four groups (normal, mild, moderate, and severe elevation) as shown in Supplementary Table 1. If liver enzymes were measured twice during this period, the levels in 2003 were used for the analysis. The dAAR scores were recently developed and validated to assess the impact of subclinical advanced liver fibrosis using age, AST, and ALT^17^. The calculating method of dAAR is presented in Supplementary Table 2.

### Infectious mortality as primary endpoint

Infectious mortality was defined as death due to infection. The NHIS database provided the date of death and the main cause of death of all individuals. Infectious mortality was evaluated from January 1, 2004 to December 31, 2015, over 12 years. The specific diagnoses of infectious mortality are presented as ICD-10 codes in Supplementary Table 3.

### Measurements as confounders for adjustment

The following information was collected as covariates for this study: demographic information (age, sex, and BMI), socioeconomic status (residence and annual income level), presence of comorbidities (underlying disability, Charlson comorbidity index, mild to moderate and severe liver disease), and lifestyle information (smoking status, alcohol consumption, and exercise frequency). Place of residence was classified into three groups (Seoul, other metropolitan cities, and other areas), and BMI was categorized into four groups (< 18.5, 18.5–24.9, 25.0–29.9, and > 30 kg/m^2^). The annual income level was categorized into five groups (0–20%, 20–40%, 40–60%, 60–80%, and 80–100%), and the underlying disability was divided into two groups (mild to moderate and severe disability groups). In South Korea, all physical disabilities must be registered in the NHIS to receive various benefits. The disabilities were categorized into six levels based on severity. The 1st (most severe) to 3rd level of disability was considered as the severe disability group, while the 4th to 6th (mildest) level was considered as the moderate disability group. Smoking status was divided into four groups: non-smoker, previous smoker, current smoker, and unknown [no-response group]). Alcohol consumption was divided into four groups (non-drinker, mild drinker, heavy drinker, and unknown [no-response group]). The mild drinker group was defined as alcohol consumption ≤ 70 g per week in males and ≤ 20 g per week in females, while the heavy drinker group was defined as alcohol consumption > 70 g per week in males and > 20 g per week in females. Exercise frequency was divided into six groups (no exercise, 1–2 times per week, 3–4 times per week, 5–6 times per week, exercise almost every day, and unknown [no-response group]). The Charlson comorbidity index was calculated using registered ICD-10 codes between 2002 and 2003, as shown in Supplementary Table 4^18^.

### Statistical analysis

The clinico-epidemiological characteristics of the participants in this study are presented as means with standard deviations for continuous variables and numbers with percentages for categorical variables. We constructed multivariable Cox regression models to investigate whether the AST, ALT, AST/ALT ratio, γ-GTP, and dAAR were associated with infectious mortality from 2004  to 2015. In this time to event analysis, infectious death was set as an event, and the survival time from January 1, 2004 to the date of death was set as duration. The four multivariable Cox models were fitted to avoid multicollinearity between the liver enzymes (AST, ALT, AST/ALT ratio, γ-GTP, and dAAR), and all covariates were included in the multivariable models for adjustment. In addition, as a sensitivity analysis, we investigated the association of elevated liver enzymes with infectious mortality from 2005–2015, not 2004–2015, to avoid reverse causation bias. This is because there was a short period of latency between elevated liver enzymes and infectious mortality in 2004^19^. Further, we performed subgroup analyses according to comorbidities of liver disease and alcohol consumption because both liver disease and alcohol consumption affect the elevation of liver enzymes^13^. The multivariable Cox regression models were also used for the subgroup analyses using the same methods as those for the main analyses. It was confirmed that there was no multicollinearity in all multivariable models involving the entire cohort, with a variance inflation factor of < 2.0. The Cox regression results are presented as hazard ratios (HRs) with 95% confidence intervals (CIs). All statistical analyses were performed using R software (version 4.0.3 with R packages, the R Project for Statistical Computing, Vienna, Austria), and *P* < 0.05 was considered statistically significant.

## Results

### Population

**Figure 1:**
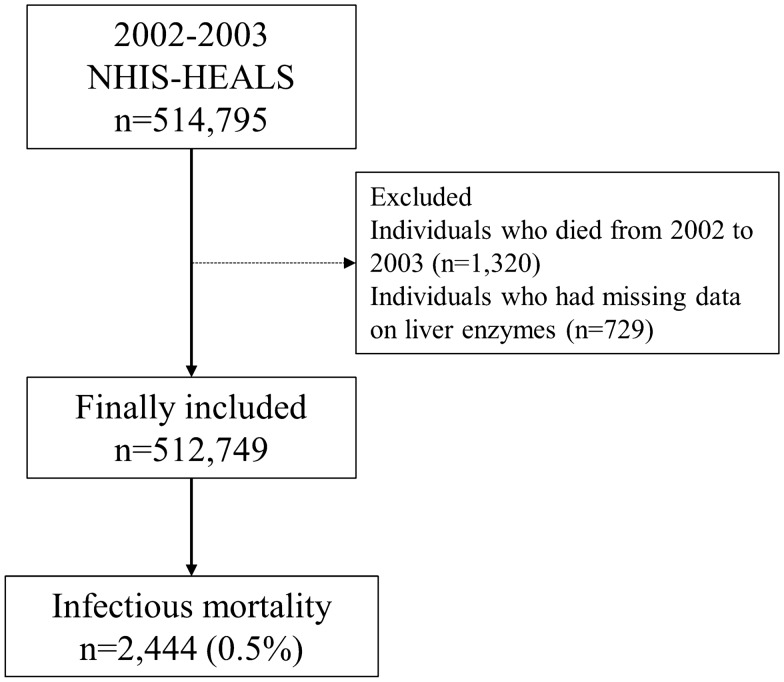
Flow chart depicting the enrolment process. NHIS-HEALS, National Health Insurance Service-National Health Screening Cohort.

A total of 514,795 individuals underwent the standardized medical examination from 2002 to 2003. Among them, 1320 individuals who died during this period and 729 individuals whose information about liver enzymes were missing were excluded. A total of 512,746 individuals were included in this study. Among them, infectious mortality occurred in 2444 individuals (0.5%), as shown in Fig. 1. The clinico-epidemiological characteristics of the study’s participants are presented in Table 1.

**Table 1:** The clinico-epidemiological characteristics of the participants (n = 512,746).

Variable	Number (%)	Mean (SD)
Age, year		53.6 (9.6)
Sex, male	277,758 (54.2)	
**Residence at diagnosis**		
Seoul (capital city)	87,993 (17.2)	
Other metropolitan city	140,239 (27.4)	
Other area	284,514 (55.5)	
**Body mass index, kg/m**^**2**^		
18.5–24.9 (normal)	320,852 (62.6)	
Below 18.5 (underweight)	11,860 (2.3)	
25.0–29.9 (overweight)	164,840 (32.1)	
Above 30.0 (obese)	14,717 (2.9)	
Unknown	477 (0.1)	
**Annual income level**		
0–20% (lowest)	80,547 (15.7)	
20–40%	69,896 (13.6)	
40–60%	80,940 (15.8)	
60–80%	107,981 (21.1)	
80–100% (highest)	173,382 (33.8)	
**Underlying disability**		
Mild to moderate	2052 (0.4)	
Severe	1245 (0.2)	
**Smoking status**		
Never smoker	331,264 (64.6)	
Previous smoker	43,501 (8.5)	
Current smoker	117,291 (22.9)	
Unknown	20,690 (4.0)	
**Alcohol consumption**		
No drink	285,927 (55.8)	
Mild drink group	89,244 (17.4)	
Heavy drink group	125,752 (24.5)	
Unknown	11,823 (2.3)	
**Exercise frequency**		
No exercise	285,668 (55.7)	
1–2 per a week	118,117 (23.0)	
3–4 per a week	47,073 (9.2)	
5–6 per a week	13,170 (2.6)	
Almost everyday	34,690 (6.8)	
Unknown	14,028 (2.7)	
Charlson comorbidity index		1.3 (1.6)
Family history of liver disease	15,459 (3.0)	
Comorbid of mild liver disease	84,427 (16.5)	
Comorbid of moderate to severe liver disease	1618 (0.3)	
**AST**		26.9 (17.7)
Normal	383,050 (74.7)	
Mild elevation	90,044 (17.6)	
Moderate elevation	36,188 (7.1)	
Severe elevation	3464 (0.7)	
**ALT**		25.9 (21.0)
Normal	387,619 (75.6)	
Mild elevation	65,905 (12.9)	
Moderate elevation	54,435 (10.6)	
Severe elevation	4787 (0.9)	
AST/ALT ratio		1.2 (0.9)
dAAR		− 0.04 (1.24)
**γ-GTP**		37.8 (54.4)
Normal	387,978 (75.7)	
Mild elevation	82,824 (16.2)	
Moderate elevation	21,080 (4.1)	
Severe elevation	20,864 (4.1)	

SD, standard deviation; AST, aspartate transaminase; ALT, alanine aminotransferase; γ-GTP, γ-glutamyl transpeptidase.

### Liver enzyme elevation and infectious mortality

**Figure 2:**
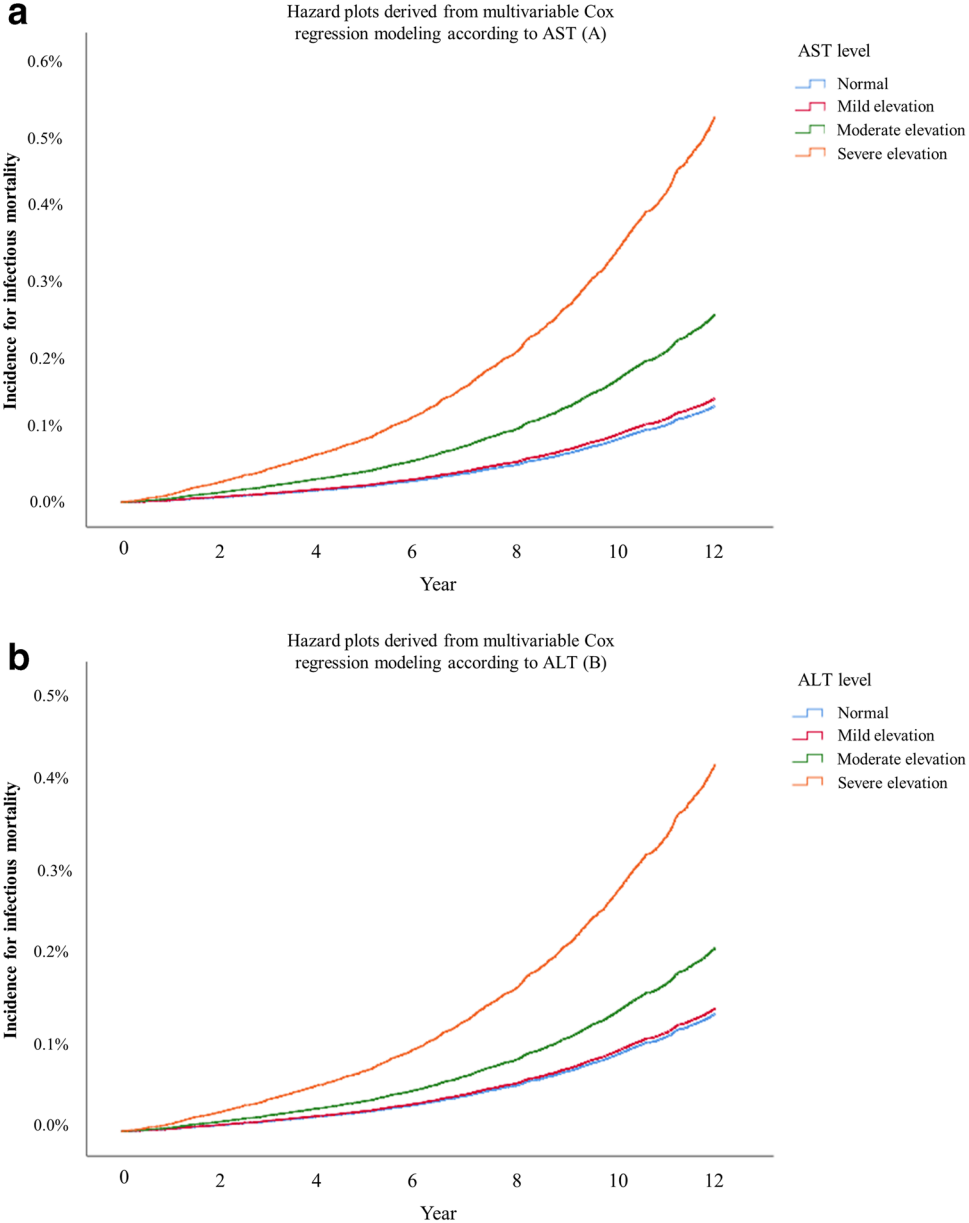

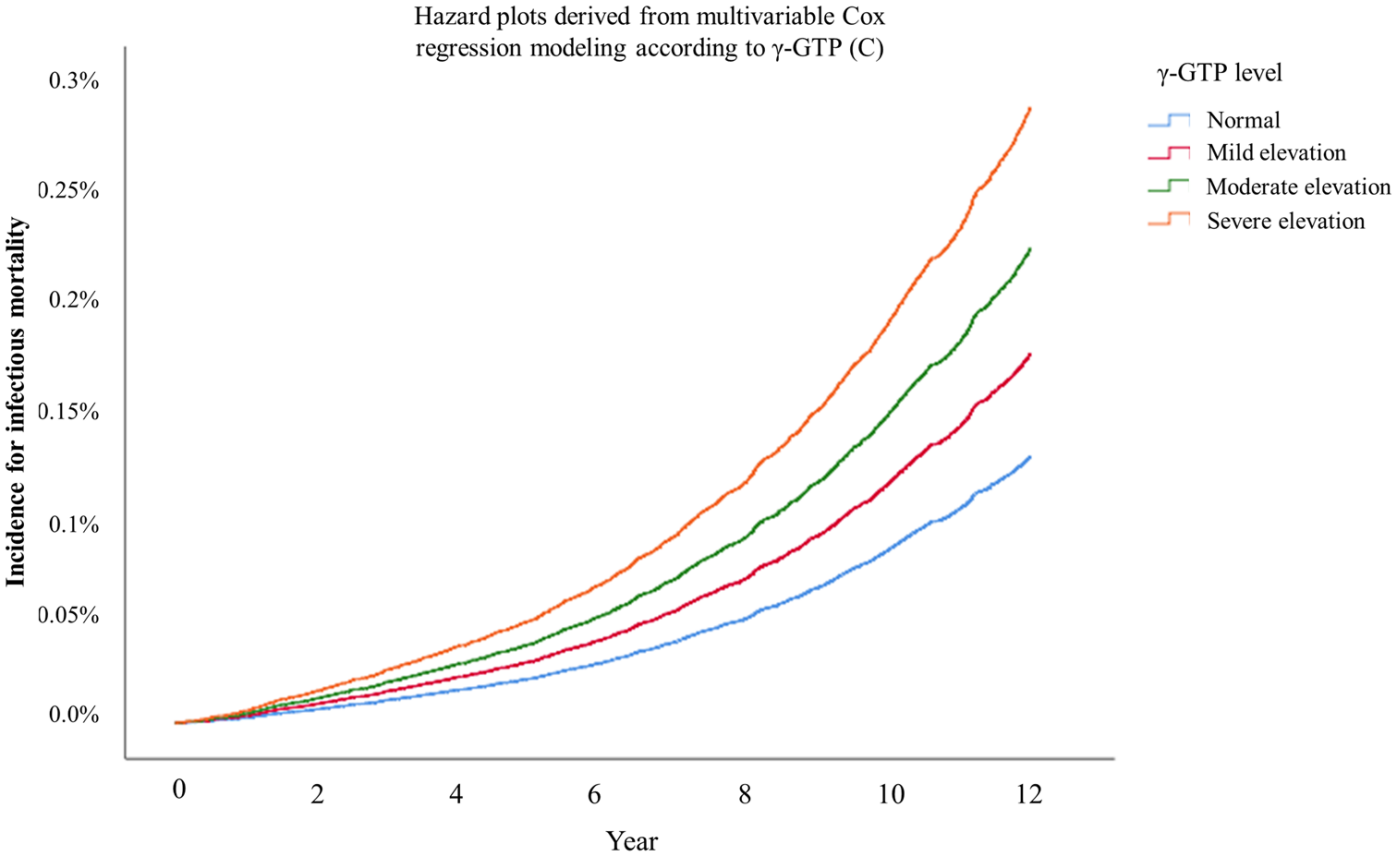
Hazard plots derived from multivariable Cox regression modeling according to AST (**a**), ALT (**b**), and γ-GTP (**c**) level elevation.

Table 2 shows the results of the multivariable Cox regression models for infectious mortality. Moderate and severe elevations of AST were associated with 1.94-fold (HR:1.94, 95% CI 1.71–2.19; *P* < 0.001) and 3.93-fold (HR: 3.93, 95% CI 3.05–5.07; *P* < 0.001) higher infectious mortality compared to that of the normal group. Similarly, moderate and severe ALT elevations were associated with 1.56-fold (HR:1.56, 95% CI 1.37–1.78; *P* < 0.001) and 3.12-fold (HR: 3.12, 95% CI 2.34–4.15; *P* < 0.001) higher infectious mortality compared to that of the normal group. Mild, moderate, and severe γ-GTP elevation group was associated with 1.38-fold (HR:1.38, 95% CI 1.23–1.55; *P* < 0.001), 1.78-fold (HR:1.78, 95% CI 1.46–2.16; *P* < 0.001), and 2.30-fold (HR:2.30, 95% CI 1.95–2.73; *P* < 0.001) higher infectious mortality compared to that of the normal group. Figure 2a–c show these trends as hazard plots, from the multivariable Cox regression models. A single-point increase in the AST/ALT ratio was associated with 1.02-fold higher infectious mortality (HR: 1.02, 95% CI 1.00–1.03; *P* < 0.001). A single-point increase in the dAAR was associated with 1.12-fold higher infectious mortality (HR: 1.12, 95% CI 1.11–1.14; *P* < 0.001). Supplementary Table 5 shows the results of sensitivity analysis regarding the multivariable Cox regression model for infectious mortality from 2005 to 2015 (not 2004–2015), and the results were similar to that of the main analyses. The results of subgroup analyses according to comorbid liver disease and alcohol consumption are presented in Tables 3 and 4. The HRs of elevation in AST, ALT, γ-GTP, and dAAR for infectious mortality tended to be higher in the liver disease group than in the no liver disease group. The HRs of elevation in AST, ALT, γ-GTP, and dAAR for infectious mortality tended to be higher in the alcohol user group than in the non-alcohol user group.

**Table 2:** Multivariable Cox regression models for infectious mortality.

Variable	Multivariable Cox model	*P*-value
	Hazard ratio (95% CI)	
**AST (model 1)**		
Normal	1	
Mild elevation	1.08 (0.97, 1.20)	0.187
Moderate elevation	1.94 (1.71, 2.19)	< 0.001
Severe elevation	3.93 (3.05, 5.07)	< 0.001
**ALT (model 2)**		
Normal	1	
Mild elevation	1.05 (0.92, 1.20)	0.505
Moderate elevation	1.56 (1.37, 1.78)	< 0.001
Severe elevation	3.12 (2.34, 4.15)	< 0.001
AST/ALT ratio (model 3)	1.02 (1.01, 1.03)	< 0.001
**γ-GTP (model 4)**		
Normal	1	
Mild elevation	1.38 (1.23, 1.55)	< 0.001
Moderate elevation	1.78 (1.46, 2.16)	< 0.001
Severe elevation	2.30 (1.95, 2.73)	< 0.001
dAAR, 1 increase	1.12 (1.11, 1.14)	< 0.001
Age, year	1.17 (1.16, 1.17)	< 0.001
Sex, male	2.12 (1.92, 2.35)	< 0.001
**Residence at diagnosis**		
Seoul (capital city)	1	
Other metropolitan city	1.18 (1.02, 1.37)	0.030
Other area	1.23 (1.08, 1.40)	0.002
**Body mass index, kg/m**^**2**^		
18.5–24.9 (normal)	1	
Below 18.5 (underweight)	2.35 (2.06, 2.69)	< 0.001
25.0–29.9 (overweight)	0.73 (0.65, 0.80)	< 0.001
Above 30.0 (obese)	1.00 (0.77, 1.28)	0.976
Unknown	0.31 (0.04, 2.19)	0.240
**Annual income level**		
0–20% (lowest)	1	
20–40%	0.91 (0.80, 1.03)	0.117
40–60%	0.84 (0.74, 0.96)	0.010
60–80%	0.74 (0.65, 0.84)	< 0.001
80–100% (highest)	0.66 (0.59, 0.74)	< 0.001
**Underlying disability**		
Mild to moderate	0.61 (0.42, 0.88)	0.008
Severe	0.72 (0.45, 1.15)	0.171
**Smoking status**		
Never smoker	1	
Previous smoker	1.34 (1.16, 1.55)	< 0.001
Current smoker	1.50 (1.35, 1.67)	< 0.001
Unknown	1.06 (0.82, 1.37)	0.648
**Alcohol consumption (frequency)**		
No drink	1	
Mild drink group	0.75 (0.66, 0.85)	< 0.001
Heavy drink group	0.84 (0.75, 0.94)	0.002
Unknown	1.00 (0.74, 1.36)	0.994
**Exercise frequency**		
No exercise	1	
1–2 per a week	0.82 (0.72, 0.93)	0.002
3–4 per a week	0.75 (0.62, 0.91)	0.003
5–6 per a week	0.72 (0.52, 1.00)	0.047
Almost everyday	0.80 (0.69, 0.93)	0.003
Unknown	1.15 (0.89, 1.49)	0.291
Charlson comorbidity index	1.12 (1.09, 1.14)	< 0.001
Family history of liver disease	0.90 (0.80, 1.01)	0.066
Comorbid of mild liver disease	1.03 (0.93, 1.15)	0.541
Comorbid of moderate to severe liver disease	1.40 (0.93, 2.10)	0.109

CI, confidence interval; AST, aspartate transaminase; ALT, alanine aminotransferase; γ-GTP, γ-glutamyl transpeptidase.

**Table 3:** Subgroup analyses according to comorbid of liver disease.

Variable	Multivariable Cox model	*P*-value
	Hazard ratio (95% CI)	
**Liver disease group (n = 85,087)**		
AST (model 1)		
Normal	1	
Mild elevation	1.15 (0.91, 1.44)	0.246
Moderate elevation	2.23 (1.80, 2.76)	< 0.001
Severe elevation	4.56 (3.19, 6.53)	< 0.001
ALT (model 2)		
Normal	1	
Mild elevation	0.97 (0.74, 1.27)	0.820
Moderate elevation	1.67 (1.34, 2.08)	< 0.001
Severe elevation	3.19 (2.12, 4.79)	< 0.001
AST/ALT ratio (model 3)	1.01 (1.00, 1.03)	0.157
γ-GTP (model 4)		
Normal	1	
Mild elevation	1.41 (1.13, 1.77)	0.003
Moderate elevation	2.22 (1.61, 3.06)	< 0.001
Severe elevation	2.45 (1.85, 3.25)	< 0.001
dAAR (model 5)	1.15 (1.12, 1.18)	< 0.001
**No Liver disease group (n = 427,659)**		
AST (model 1)		
Normal	1	
Mild elevation	1.06 (0.94, 1.20)	0.358
Moderate elevation	1.82 (1.56, 2.12)	< 0.001
Severe elevation	3.52 (2.45, 5.07)	< 0.001
ALT (model 2)		
Normal	1	
Mild elevation	1.08 (0.93, 1.26)	< 0.333
Moderate elevation	1.49 (1.27, 1.76)	< 0.001
Severe elevation	2.94 (1.96, 4.41)	< 0.001
AST/ALT ratio (model 3)	1.03 (1.02, 1.04)	< 0.001
γ-GTP (model 4)		
Normal	1	
Mild elevation	1.37 (1.20, 1.57)	< 0.001
Moderate elevation	1.58 (1.23, 2.03)	< 0.001
Severe elevation	2.28 (1.85, 2.81)	< 0.001
dAAR (model 5)	1.11 (1.09, 1.13)	< 0.001

CI, confidence interval; AST, aspartate transaminase; ALT, alanine aminotransferase; γ-GTP, γ-glutamyl transpeptidase.

**Table 4:** Subgroup analyses according to alcohol consumption.

Variable	Multivariable Cox model	*P*-value
	Hazard ratio (95% CI)	
**No alcohol use (n = 285,927)**		
AST (model 1)		
Normal	1	
Mild elevation	1.10 (0.97, 1.26)	0.127
Moderate elevation	1.94 (1.64, 2.30)	< 0.001
Severe elevation	3.83 (2.59, 5.65)	< 0.001
ALT (model 2)		
Normal	1	
Mild elevation	1.05 (0.90, 1.23)	0.521
Moderate elevation	1.39 (1.16, 1.67)	< 0.001
Severe elevation	2.91 (1.93, 4.41)	< 0.001
AST/ALT ratio (model 3)	1.04 (1.02, 1.06)	< 0.001
γ-GTP (model 4)		
Normal	1	
Mild elevation	1.60 (1.38, 1.87)	< 0.001
Moderate elevation	1.98 (1.45, 2.72)	< 0.001
Severe elevation	2.18 (1.60, 2.97)	< 0.001
dAAR (model 5)	1.11 (1.10, 1.13)	< 0.001
**Alcohol use group (n = 214,996)**		
AST (model 1)		
Normal	1	
Mild elevation	1.03 (0.83, 1.27)	0.819
Moderate elevation	1.92 (1.60, 2.30)	< 0.001
Severe elevation	4.19 (3.00, 5.85)	< 0.001
ALT (model 2)		
Normal	1	
Mild elevation	1.01 (0.78, 1.31)	0.953
Moderate elevation	1.76 (1.45, 2.15)	< 0.001
Severe elevation	3.63 (2.44, 5.40)	< 0.001
AST/ALT ratio (model 3)	1.02 (1.01, 1.03)	< 0.001
γ-GTP (model 4)		
Normal	1	
Mild elevation	1.11 (0.93, 1.33)	0.243
Moderate elevation	1.61 (1.79, 2.71)	< 0.001
Severe elevation	2.20 (1.79, 2.71)	< 0.001
dAAR (model 5)	1.18 (1.13, 1.23)	< 0.001

CI, confidence interval; AST, aspartate transaminase; ALT, alanine aminotransferase; γ-GTP, γ-glutamyl transpeptidase.

## Discussion

This population-based cohort study showed that elevated liver enzymes (AST, ALT, AST/ALT ratio, γ-GTP, and dAAR) were associated with a higher risk of infectious mortality among the South Korean adult population. This association was most evident in the elevation of AST. Specifically, our results suggested that the elevation of AST, ALT, and γ-GTP above 40 U was associated with a higher risk of infectious mortality. In subgroup analyses, this association was more evident in the existing liver disease and alcohol user groups. This is the first study to report an increased risk of infectious mortality due to abnormal liver function among the adult population.

We found that approximately 25% of the adult population had elevated liver enzyme levels between 2002 and 2003. It was estimated that about 9% of asymptomatic patients had elevated enzyme levels on standard liver function tests in the United States^12^. Our cohort included 16.8% of the patients who were diagnosed with mild, moderate, and severe liver disease; thus, 25% of the cohort showing elevated liver enzyme in our study can be considered acceptable.

A previous study reported that advanced liver disease generally increases the risk of infection, and this phenomenon is known as cirrhosis-associated immune dysfunction^20^. Cirrhosis-associated immune dysfunction was defined as syndromic abnormalities of the immune function, characterized by immunodeficiency and systemic inflammation^20^. As an immune defense mechanism, the liver prevents the systemic spread of microbial and dietary antigens entering through the gastrointestinal tract^21^. This function is offset by immune tolerance to non-pathogenic exogenous material^22^. Another mechanism is the synthesis of soluble molecules that are essential for an effective immune response^5^. However, cirrhosis disrupts the cellular organization of the liver and reduces the ability of hepatocytes to synthesize proteins^20^. These cirrhosis-induced liver injuries compromise the immune function of the liver by damaging the reticuloendothelial system and synthesis of innate immune proteins. These damaged reticuloendothelial systems due to liver cirrhosis have been identified as risk factors for bacterial infection and lower survival rates^23^. In addition to the previous reports regarding cirrhosis-associated immune dysfunction and risk of infection, our study reported that infectious mortality might be increased in the adult population with abnormal liver function. In other words, not only advanced liver diseases, such as liver cirrhosis but also adult populations with mild liver disease might have a higher risk for infectious mortality.

The results regarding elevated γ-GTP and increased risk of infectious mortality are also important. γ-GTP is found in hepatocytes and biliary epithelial cells and is a very sensitive marker of hepatobiliary disease^24^, although it has lower specificity than AST and ALT for diagnosing liver disease^24^. The γ-GTP level is known to increase more consistently, with high sensitivity than AST and ALT among patients with all types of anicteric non-alcoholic chronic liver disease^25^. Moreover, the serum γ-GTP level for assessment of liver fibrosis or cirrhosis combined with platelet count is an important biomarker in patients with chronic hepatitis B infection^26^. Although we did not evaluate the γ-GTP to platelet count ratio due to the lack of platelet count data in our database, our results suggest that γ-GTP elevation might significantly reflect abnormal liver function and can be a predictive factor for higher infectious mortality among individuals with abnormal liver function.

In the subgroup analyses, our study showed that the impact of elevation in liver enzymes on increased infectious mortality was more evident in individuals with pre-existing liver disease and alcohol users. In general, the severity of liver disease correlates with increased liver enzyme levels^11^. In the population with chronic liver disease or alcohol users, the liver enzymes might directly reflect liver function. Elevation of liver enzymes is also seen in non-hepatic diseases, such as congestive heart failure, celiac disease, thyroid disorders, or striated muscle disease^13^. However, there are few reports on the relationship between the risk of infection and elevated liver enzyme levels in those without liver disease among the adult population; therefore, further research is necessary.

Interestingly, we used dAAR to evaluate the impact of subclinical advanced liver fibrosis using age, AST, and ALT according to a recent study^17^. In the recent study^17^, the dAAR score provides a method to stratify easily the adult population regarding risk for incident severe liver disease. Furthermore, the dAAR score can be used as a screening tool for the unselected general population and as a trigger for further liver evaluations in the future. However, currently, it is a new concept in screening for liver function; therefore, further research is required to evaluate the clinical usefulness of dAAR.

Our study had some limitations. First, some important variables, such as platelet count and bilirubin, were not included in the study because the NHIS database did not contain these data. Therefore, the impact of the AST to platelet ratio index or albumin-bilirubin score on infectious mortality could not be analyzed. Second, multivariable adjustment only adjusted for known confounders, and there might be unmeasured confounders that might affect the results. Third, we used ICD-10 codes to define liver disease and calculate the Charlson comorbidity index; however, the actual underlying diseases might differ with registered ICD-10 codes in this study. For instance, if individuals with liver disease did not visit outpatient clinics due to mild symptoms or poor access to healthcare utilization, they were not registered as having liver disease in the NHIS database in this study. Lastly, the lifestyle information was collected using a questionnaire, and there might be a selection bias due to non-response to the surveys in standardized medical examination^27^.

In conclusion, this population-based cohort study showed that elevated liver enzymes (AST, ALT, AST/ALT ratio, γ-GTP, and dAAR) were associated with increased infectious mortality among South Korean adults. Specifically, our results suggest that the elevation of AST, ALT, and γ-GTP above 40 U were significantly associated with infectious mortality. Therefore, screening and optimal management of adult populations with elevated enzymes are clinically important for preventing infectious mortality.

## Supplementary Information

Supplementary Table S1.

Supplementary Table S2.

Supplementary Table S3.

Supplementary Table S4.

Supplementary Table S5.

## Data Availability

The datasets used and/or analyzed during the current study are available from the corresponding author upon reasonable request.
