# Supplementary material for: Long-term mortality due to infection associated with elevated liver enzymes: a population-based cohort study
Source: Sci Rep. 2021 Jun 14;11:12490. doi: 10.1038/s41598-021-92033-1 (PMC8203630; doi:10.1038/s41598-021-92033-1)
Supplement: Supplementary file 1 — Supplementary Table S1. [file 41598_2021_92033_MOESM1_ESM.docx]

Supplementary Table 1. Definition of elevation in liver enzyme

| Elevation in liver enzyme | AST | ALT | GGT |
| --- | --- | --- | --- |
| Normal | ≤ 33 IU in male  ≤ 25 IU in female | ≤ 33 IU in male  ≤ 25 IU in female | ≤ 40 IU |
| Mild elevation | 33-40 IU in male  25-40 IU in female | 33-40 IU in male  25-40 IU in female | 40-80 IU |
| Moderate elevation | 40-100 IU | 40-100 IU | 80-120 IU |
| Severe elevation | > 100 IU | > 100 IU | > 120 IU |

AST, Aspartate transaminase; ALT, alanine aminotransferase; γ-GTP, γ-glutamyl transpeptidase
