# Supplementary material for: Long-term mortality due to infection associated with elevated liver enzymes: a population-based cohort study
Source: Sci Rep. 2021 Jun 14;11:12490. doi: 10.1038/s41598-021-92033-1 (PMC8203630; doi:10.1038/s41598-021-92033-1)
Supplement: Supplementary file 2 — Supplementary Table S2. [file 41598_2021_92033_MOESM2_ESM.docx]

**Supplementary Table 2. The dAAR score equation**

AGE = age in years

ALT = alanine aminotransferase (U/L)

astalt = ratio of AST to ALT (aspartate aminotransferase / alanine aminotransferase)

R software

dataset = name of the R data

dataset$riskscore1 <- -10.129915+0.039811813*dataset$AGE+0.25387407* dataset$ALT-0.0023607234*pmax(dataset$ALT-11,0)^3+0.0079492072*pmax(dataset$ALT-17,0)^3-0.0076811579*pmax(dataset$ALT-22,0)^3+0.0021985068*pmax(dataset$ALT-30,0)^3-0.00010583268*pmax(dataset$ALT-58,0)^3+3.5333535* dataset$astalt-7.3473709*pmax(dataset$astalt-0.63,0)^3+32.911587*pmax(dataset$astalt-0.92,0)^3-44.937707*pmax(dataset$astalt-1.14,0)^3+21.786619*pmax(dataset$astalt-1.41,0)^3-2.4131284*pmax(dataset$astalt-2.13,0)^3

MS Excel

=(-10,129915)+(0,039811813*AGE)+(0,25387407*ALT)-(0,0023607234*MAX(0;ALT-11)^3)+(0,0079492072*MAX(0;ALT-17)^3)-(0,0076811579*MAX(0;ALT-22)^3)+(0,0021985068*MAX(0;ALT-30)^3)-(0,00010583268*MAX(0;ALT-58)^3)+(3,5333535*(astalt))-(7,3473709*MAX(0;astalt-0,63)^3)+(32,911587*MAX(0;astalt-0,92)^3)-(44,937707*MAX(0;astalt-1,14)^3)+(21,786619*MAX(0;astalt-1,41)^3)-(2,4131284*MAX(0;astalt-2,13)^3)

STATA

generate riskscore1=-10.129915+ 0.039811813*AGE+0.25387407*ALT-0.0023607234*max((ALT-11),0)^3+ 0.0079492072*max((ALT-17),0)^3-0.0076811579*max((ALT-22),0)^3+0.0021985068*max((ALT-30),0)^3- 0.00010583268*max((ALT-58),0)^3+3.5333535*astalt-7.3473709*max((astalt-0.63),0)^3+ 32.911587*max((astalt-0.92),0)^3-44.937707*max((astalt-1.14),0)^3+21.786619*max((astalt-1.41),0)^3-2.4131284*max((astalt-2.13),0)^3

SAS

riskscore1=-10.129915+0.039811813*AGE+0.25387407*ALT-0.0023607234*max((ALT-11),0)**3+0.0079492072*max((ALT-17),0)**3-

0.0076811579*max((ALT-22),0)**3+0.0021985068*max((ALT-30),0)**3-

0.00010583268*max((ALT-58),0)**3+3.5333535*astalt-7.3473709*max((astalt-0.63),0)**3+32.911587*max((astalt-0.92),0)**3-44.937707*max((astalt-1.14),0)**3+21.786619*max((astalt-1.41),0)**3-

2.4131284*max((astalt-2.13),0)**3;
