# Supplementary material for: Long-term mortality due to infection associated with elevated liver enzymes: a population-based cohort study
Source: Sci Rep. 2021 Jun 14;11:12490. doi: 10.1038/s41598-021-92033-1 (PMC8203630; doi:10.1038/s41598-021-92033-1)
Supplement: Supplementary file 5 — Supplementary Table S5. [file 41598_2021_92033_MOESM5_ESM.docx]

**Supplementary Table 5.** **Multivariable Cox regression model for infectious mortality during 2005-2015**

| Variable | | Multivariable Cox model | *P*-value |
| --- | --- | --- | --- |
|  |  | Hazard Ratio (95% CI) |  |
| AST (model 1) | |  |  |
|  | Normal | 1 |  |
|  | Mild elevation | 1.07 (0.96, 1.19) | 0.237 |
|  | Moderate elevation | 1.91 (1.69, 2.17) | <0.001 |
|  | Severe elevation | 3.99 (3.09, 5.16) | <0.001 |
| ALT (model 2) | |  |  |
|  | Normal | 1 |  |
|  | Mild elevation | 1.05 (0.91, 1.20) | 0.517 |
|  | Moderate elevation | 1.57 (1.38, 1.80) | <0.001 |
|  | Severe elevation | 3.20 (2.39, 4.27) | <0.001 |
| AST/ALT ratio (model 3) | | 1.02 (1.01, 1.03) | <0.001 |
| γ-GTP (model 4) | |  |  |
|  | Normal | 1 |  |
|  | Mild elevation | 1.38 (1.23, 1.56) | <0.001 |
|  | Moderate elevation | 1.76 (1.44, 2.15) | <0.001 |
|  | Severe elevation | 2.30 (1.94, 2.73) | <0.001 |
| dAAR (model 5) | | 1.15 (1.07, 1.23) | <0.001 |

CI, confidence interval; AST, Aspartate transaminase; ALT, alanine aminotransferase; γ-GTP, γ-glutamyl transpeptidase
